# Seizures in patients with *IDH*-mutated lower grade gliomas

**DOI:** 10.1007/s11060-022-04158-6

**Published:** 2022-10-18

**Authors:** Louise Carstam, Isabelle Rydén, Asgeir Store Jakola

**Affiliations:** 1 Department of Neurosurgery, Sahlgrenska University Hospital, Blå Stråket 5, 41345 Gothenburg, Sweden; 2 Institute of Neuroscience and Physiology, Sahlgrenska Academy, University of Gothenburg, Göteborg, Sweden

**Keywords:** Seizures, Epilepsy, Lower grade glioma, *IDH*-mutation, 1p19q, EOR

## Abstract

**Purpose:**

Most patients with Lower Grade Gliomas (LGG) present with epileptic seizures. Since the advent of molecular diagnostics, more homogenous sub-entities have emerged, including the isocitrate dehydrogenase-mutated *(IDH*-mutated) astrocytomas and 1p19q-codeleted oligodendrogliomas. We aimed to describe the occurrence of seizures in patients with molecularly defined LGG pre- and postoperatively and to analyze factors affecting seizure status postoperatively.

**Methods:**

A population-based cohort of 130 adult patients with *IDH*-mutated WHO grade 2 or 3 astrocytomas and oligodendrogliomas was assessed pertaining to seizure burden before and after surgery.

**Results:**

Fifty-four (79.4%) patients with astrocytoma and 45 (72.6%) patients with oligodendroglioma had a history of seizures before surgery. At 12 months postoperatively, 51/67 (76.1%) patients with astrocytoma and 47/62 (75.8%) patients with oligodendrogliomas were seizure free. In a multivariable logistic regression analysis, lower extent of resection (EOR) (OR 0.98; 95% CI 0.97–1.00, p = 0.01) and insular tumor location (OR 5.02; 95% CI 1.01–24.87, p = 0.048) were associated with presence of seizures within 1 year postoperatively in the entire LGG cohort. In sub-entities, EOR was in a similar manner associated with seizures postoperatively in astrocytomas (OR 0.98; 95% CI 0.96–0.99, p < 0.01) but not in oligodendrogliomas (p = 0.34).

**Conclusion:**

Our results are well in line with data published for non-molecularly defined LGG with a large proportion of patients being seizure free at 1 year postoperative. Better seizure outcome was observed with increased EOR in astrocytomas, but this association was absent in oligodendrogliomas.

**Supplementary Information:**

The online version contains supplementary material available at 10.1007/s11060-022-04158-6.

## Introduction

Lower grade gliomas (LGG) are slow-growing, infiltrating tumors that often cause epileptic seizures as the initial symptom [1]. The patients are young to middle-aged adults, typically in the middle of their careers and family lives. Even though rarely marked by overt neurological deficits from the tumors, epileptic seizures and effects from antiepileptic drugs may exert strong influence over the patient´s everyday life or ability to work and the unpredictable nature of seizures may provoke fear and loss of control. Epileptic seizure burden is one of the factors that affect quality of life the most in patients with LGG [2, 3]

Earlier works on LGG and epilepsy are largely from the pre-molecular era, where some included both WHO grade 1 and 2 tumors [4], while yet other reports analyze *IDH*-mutated (*IDH*-mut) and *IDH*-wildtype (*IDH*-wt) morphological grade 2 gliomas together. [5–7]

Molecular characterization of LGG has been found to be of major clinical importance and is since 2016 part of the WHO diagnostic criteria [8]. We now consider the *IDH*-mut the hallmark of the true LGG [8, 9]. With better classification, several authors note that the clinical importance of the distinction between WHO grade 2 and grade 3 *IDH*-mut LGG is of lesser importance in prognostication [10, 11]. In the light of these updates, it makes sense analyzing the more homogenous group of purely *IDH*-mut glioma with respect to epileptic seizures. Further, data for astrocytomas and oligodendrogliomas should, when possible, be presented separately since these tumor subtypes differ in several aspects. The objective of the present study was to describe the pre- and postoperative seizure burden in *IDH*-mut diffuse grade 2 and 3 gliomas with and without 1p19q-codeletion. In addition, we wanted to analyze potential factors affecting postoperative seizure outcome for these patients.

## Methods

### Study population

Adult patients (≥ 18 years) with LGG within the western healthcare region in Sweden with a first-time surgery (resective or biopsy) between January 2010 and December 2020 were consecutively identified. Patients with a histomolecular diagnosis of *IDH*-mutated WHO grade 2 or 3 astrocytoma or oligodendroglioma were included in the study.

### Data collection

Medical records and radiological images were used to identify patient, tumor, and treatment characteristics. Preoperative use of AEDs (AED preop) was defined as using at least one AED at the day of surgery. Importantly, primary prophylaxis with AEDs perioperatively was not used in the studied clinical setting. Time to surgery was calculated from the diagnostic scan date. Eloquent tumor location was assessed according to Chang et al. [12] For volume segmentation, semi-automatic segmentations with the software 3DSlicer version 4.6.2 or newer was made as described earlier [11, 13]. For biopsied patients, residual tumor volume was registered as equal to the preoperative tumor volume if no postoperative segmentation was made. Gross total resection (GTR) was defined as a residual tumor volume of 0 ml on T2/FLAIR-weighted postoperative MRI.

The pathological evaluation was initially made according to the WHO criteria valid at the time of surgery and when needed, reclassified as previously described so that *IDH*-mutation and 1p/19q codeletion status were known for all patients. [14]

### Seizure outcome

Medical records were searched from the time of tumor discovery up to 2 years after surgery, to identify events of seizures. The most common way of reporting seizure outcome in the LGG research is through the Engel classification system [15], either applying Engel class 1 as seizure freedom postoperatively [6, 16, 17] or referring to class 1a only as seizure freedom [5, 18, 19]. The Engel class 1 definition (allowing for example “non-disabling” or a few disabling seizures) can be argued to be too permissive to be a suitable outcome measurement for the current population with their often-better preoperative seizure situation and shorter follow-up than for the patients undergoing pure epilepsy surgery, for whom the classification system was originally created [15]. In the present study, we chose to define seizure freedom as *entirely free from seizures or aura* (Engel class 1a/ILAE definition of seizure freedom) [20] with an only exception for seizures within the first week after surgery, ascribing these to the surgical trauma.

### Statistical analyses

All analyses were done with SPSS, version 28 or newer (Chicago, IL, USA). Statistical significance level was set to p < 0.05. All tests were two-sided. Central tendencies are presented as means ± SD, or median with first and third quartile if skewed. Dichotomous data were analyzed with Fisher Exact test. Uni- and multivariable logistic regression analyses were performed. Epileptic seizures within the first year after surgery were set as outcome variable. Independent variables were chosen based on previous studies and presumed clinical relevance [5–7, 16–19, 21–23]. Variables associated at the p < 0.1 level in unadjusted analyses were entered into multivariable regression models. In the case of significant correlation between two continuous co-variates, only the one with the stronger association in univariable analysis was entered into the final adjusted model.

Response operative characteristics (ROC) curves were made to illustrate the predictive capacity of extent of resection (EOR) for seizure outcome. [24]

## Results

In our cohort of 130 patients with *IDH*-mut LGG, 68 had astrocytomas (without 1p19q-codeletion) and 62 had oligodendrogliomas (with 1p19q-codeletion). The distribution over WHO grade was 66 WHO grade 2 tumors and 64 WHO grade 3 tumors (50.8 vs 49.2%). These and other characteristics divided upon tumor subtypes are shown in Table 1.

**Table 1:** Tumor-, patient and treatment characteristics for all LGG and divided by tumor subtype. N = 130

	All LGG N = **130**	Astrocytomas N = **68**	Oligodendrogliomas N = **62**
Age median (Q1:Q3)	42.0 (35.0:54.3)	36.5 (31.0:51.0)	46.0 (39.8:58.0)
Female sex n (%)	57 (43.8)	31 (45.6)	26 (41.9)
History of seizures preop n (%)	99 (76.2)	54 (79.4)	45 (72.6)
AED preop n (%)	84 (64.6)	46 (67.6)	38 (61.3)
KPS score ≤ 80 n (%)	51 (39.2)	27 (39.7)	24 (38.7)
Preop motor deficit n (%)	9 (6.9)	5 (7.3)	4 (6.5)
Main location n (%)			
Frontal	81 (62.3)	37 (54.4)	44 (71.0)
Temporal	24 (18.5)	18 (26.5)	6 (9.7)
Parietal	15 (11.5)	10 (14.7)	5 (8.1)
Insular	9 (6.9)	3 (4.4)	6 (9.7)
Occipital	1 (0.8)	0 (0.0)	1 (1.6)
Eloquence, n (%)	82 (63.6)	49 (72.1)	33 (54.1)
Missing	1		1
Motor eloquence, n (%)	39 (30.2)	23 (33.8)	16 (26.2)
Missing	1		1
Time to surgery (months) median (Q1:Q3)	1.3 (0.7:2.9)	1.2 (0.6:2.5)	1.3 (0.8:3.5)
Missing	5	2	3
Intra-op motor mapping or awake surgery n (%)	38 (29.0)	17 (25.0)	21 (33.3)
Type of surgery			
Biopsy, n (%)	9 (6.9)	6 (8.8)	3 (4.8)
Resection, n (%)	121 (93.1)	62 (91.2)	59 (95.2)
Tumor volume preop in cm^3^; median (Q1:Q3)	55.2 (28.4:105.3)	59.8 (30.7:107.4)	54.3 (25.8:93.9)
Missing	1		1
Tumor residual volume postop^**a**^ in cm^3^; median (Q1:Q3)	8.6 (1.3:29.4)	8.9 (1.4:43.1)	8.0 (1.0:23.8)
Missing	3	2	1
EOR^**a**^ median (Q1:Q3)	85.4 (57.3:96.6)	85.1 (52.1:94.7)	87.4 (58.9:98.6)
GTR n (%)	25 (19.5)	11 (16.4)	14 (23.0)
Missing	2	1	1
Motor deficit postop, n (%)	32 (24.6)	15 (22.1)	17 (27.4)
WHO Grade n (%)			
2	66 (50.8)	34 (50.0)	32 (51.6)
3	64 (49.2)	34 (50.0)	30 (48.4)
Radiotherapy within one year after surgery n (%)	91 (70)	53 (77.9)	38 (61.3)
Chemotherapy within one year after surgery n (%)	93 (71.5)	52 (76.5)	41 (66.1)
Seizures within 12 months postoperatively^**b**^ n (%)	31 (24.0)	16 (23.9)	15 (24.2)
Missing	1	1	
Seizures within 24 months postoperatively^**b**^ n (%)	41 (32.5)	20 (30.8)	21 (34.4)
Missing	4	3	1
New onset seizures within 12 months n (%)	**N = 31**^**c**^ 4 (12.9)	**N = 14**^**c**^ 3 (21.4)	**N = 17**^**c**^ 1 (5.9)
Missing	0	0	0
New onset seizures within 24 months postop (%)	**N = 31**^**c**^ 7 (24.1)	**N = 14**^**c**^ 3 (23.1)	**N = 17**^**c**^ 4 (25.0)
missing	2	1	1

*EOR* Extent of resection, *KPS* Karnofsky performance status, GTR Gross total resection

^a^EOR and residual tumor volume calculated for the entire cohort (both resections and biopsies)

^b^Seizures first postoperative week excluded

^c^N here denotes the initial number of patients who had not had seizures before surgery

The bolding of "N" in the end of the table was just used to draw attention to the fact that the numbers of patients for these analyses differ from those in the previous analyses. The bolding could be removed

### Seizures

#### Pre-operatively

**Fig. 1:**
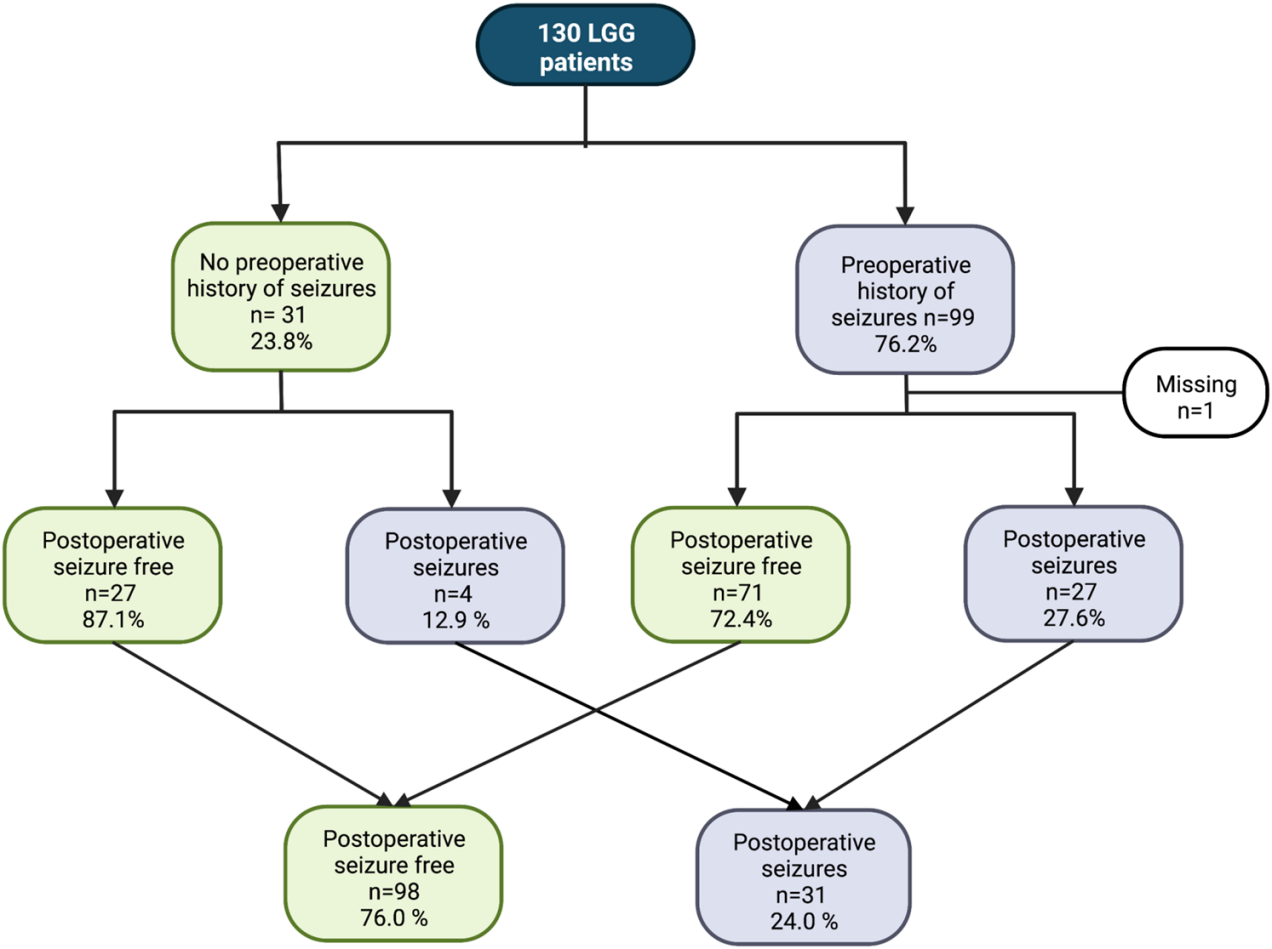
Seizure status at one year after surgery in LGG patients with and without preoperative seizures

Out of the 130 patients, 99 (76.2%) had a history of at least one epileptic seizure before surgery (Fig. 1). There was no statistically significant difference in pre-operative seizure status over tumor subtype; 79.4% (54/68) of the astrocytoma patients, and 72.6% (45/62) of the oligodendroglioma patients had experienced pre-operative seizures (p = 0.41).

#### Post-operatively

At 12 months after surgery 98/129 patients (76.0%) were entirely seizure free (Fig. 1), with an even distribution over tumor subtype (p = 1.00). After 24 months, an additional 10 patients had experienced at least one seizure, resulting in an overall seizure freedom since surgery of 67.5% (85/126). No statistically significant differences were seen over tumor subtype (p = 0.71).

### Factors affecting seizure outcome after surgery

In the univariable analyses, we found that an increased EOR was correlated with better seizure outcome, while preoperative motor-deficits and insular tumor location was associated with increased risk for post-operative seizures (Table 2). In the multivariable analysis, only insular tumor location and EOR remained significant predictors of seizure outcome postop (Table 2).

**Table 2:** Predictors for postoperative epileptic seizures within 12 months after surgery in LGG patients according to unadjusted and adjusted analysis. N = 129

Variable		Univariable analysis			Multivariable analysis		
		Unadjusted odds ratio	95% CI	p-value	Adjusted odds ratio	95% CI	p-value
Sex	Female	1 (ref)					
	Male	0.65	0.29–1.46	0.29			
Age	per year	0.99	0.95–1.02	0.35			
Preop tumor volume	per cm^3^	1.00	1.00–1.01	0.15			*
Preop motor deficit	No	1 (ref)					
	Yes	4.52	1.13–18.05	**0.03**	3.96	0.92–17.03	0.06
1p19q-codeletion	No	1 (ref)					
	Yes	1.02	0.45–2.28	0.97			
WHO grade	Grade 2	1 (ref)					
	Grade 3	0.69	0.31–1.57	0.38			
Location of tumor	Frontal	1 (ref)					
	Temporal	0.74	0.22–2.46	0.62	0.61	0.17–2.20	0.45
	Insular	6.18	1.34–28.48	**0.02**	5.02	1.01–24.87	**0.048**
	Other	1.54	0.48–4.99	0.47	1.35	0.40–4.60	0.63
Residual tumor volume	per cm^3^	1.01	1.00–1.02	0.06			*
EOR	per unit	0.98	0.97–0.99	** < 0.01**	0.98	0.97–1.00	**0.01**
Chemotherapy within 12 months postop	No	1 (ref)					
	Yes	1.19	0.49–2.87	0.71			
Radiotherapy within 12 months postop	No	1 (ref)					
	Yes	1.33	0.54–3.31	0.54			

*EOR* Extent of resection

*Not included in multivariable analysis due to significant correlation with EOR

Bold figures indicate statistical significance at the p<0.05-level

A corresponding sub-analysis was performed for astrocytomas (non-1p1q-codel) and oligodendrogliomas (1p19q-codel) separately.

In the multivariable analysis with preoperative motor deficit and EOR in astrocytomas only, EOR was the sole significant predictor of seizure outcome (p < 0.01) (Supplementary Table 1).

For oligodendrogliomas, no significant correlations were detected in multivariable analysis (Supplementary Table 2).

**Fig. 2:**
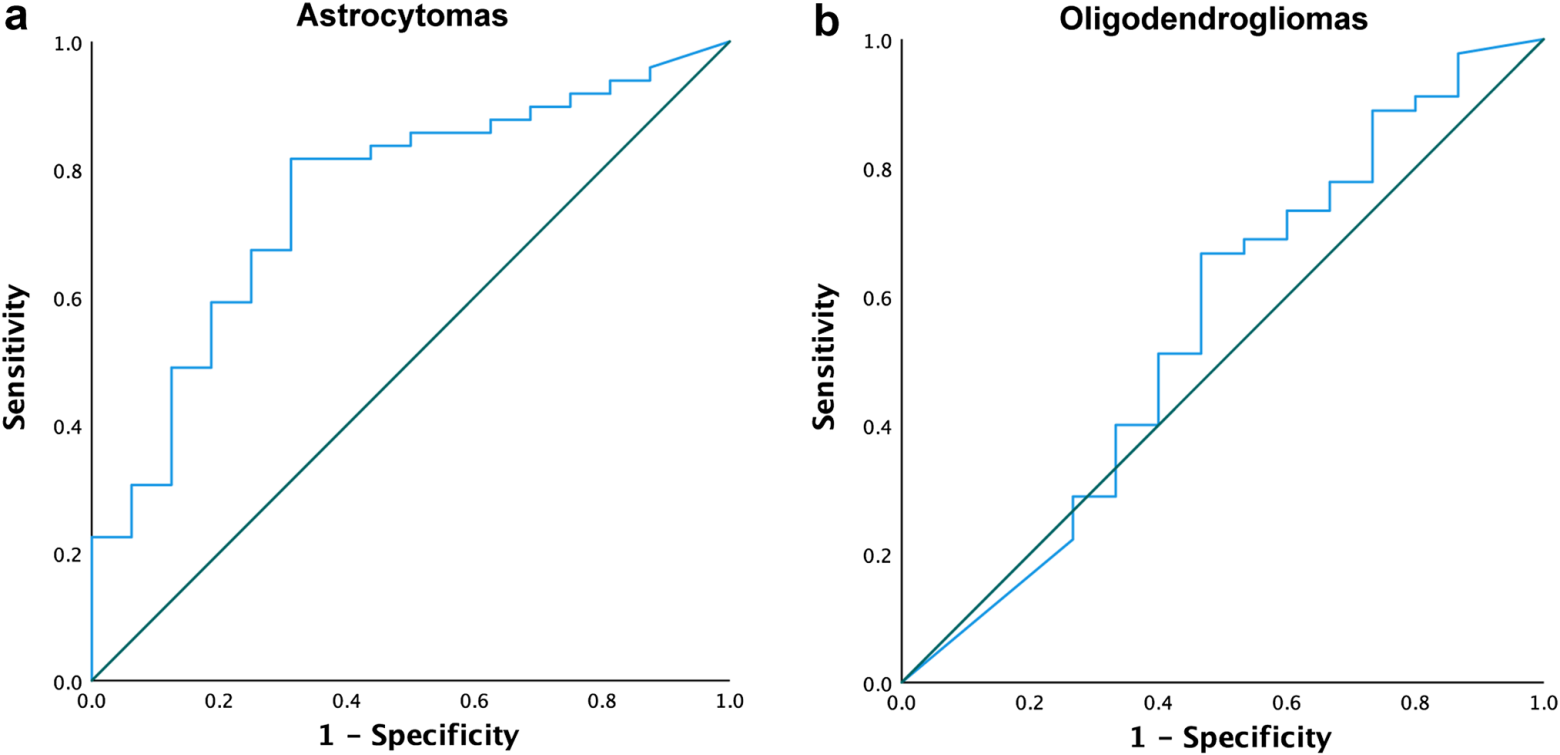
A receiver operating characteristic (ROC) curve for EOR and seizure freedom at one year after surgery. Patients with astrocytomas presented in (**a**), with an AUC of 0.75 indicating a moderate ability of EOR to predict seizure outcome. Patients with oligodendrogliomas presented in (**b**), with an AUC of 0,55 indicating a poor predictive ability. *EOR*  extent of resection, *AUC* area under the curve

A ROC curve for EOR and seizure freedom was made for astrocytomas with an area under the curve (AUC) of 0.75 (95% CI 0.62–0.89, p = 0.002) (Fig. 2a). A corresponding curve for oligodendrogliomas illustrates a lack of significant association between EOR and seizure outcome (AUC 0.55; 95% CI 0.37–0.74, p = 0.54) (Fig. 2b).

Among the 11 astrocytoma patients without any residual tumor (GTR), all were seizure free, whereas 4/14 oligodendroglioma patients with GTR had experienced at least one seizure at 1 year postoperatively.

## Discussion

This is to our knowledge the first study of epileptic seizures and seizure outcome in molecularly subtyped, purely *IDH*-mut LGG.

We found that approximately three out of four patients had experienced at least one epileptic seizure before surgery, a large majority of whom were on AEDs at the time of surgery. At 1 year postoperative, three out of four patients were seizure free. We saw no differences in seizure occurrence pre- or postoperatively between patients with 1p19q-codeleted tumors and astrocytomas. A significant correlation found between EOR and seizure freedom in astrocytomas was, quite surprisingly, absent in oligodendrogliomas.

### Seizures at diagnosis

In the entire cohort, the incidence of pre-operative seizures was well in accord with the around 70–80% reported in earlier, mainly histological, LGG publications. [5–7, 25, 26]

An even higher incidence in an all *IDH*-mut cohort could have been suspected since epilepsy seems to be more common at diagnosis in *IDH*-mut tumors than in their *IDH*-wt counterparts [27–30]. However, low grade tumors like ganglioglioma and DNET that are even more epileptogenic than LGG may have affected results in older studies. [1]

Chang et al. in 2008 found that histologically defined oligodendroglioma and oligoastrocytoma subtypes were significantly more likely to be associated with seizures than astrocytomas [6]. However, in analyses where oligodendrogliomas are categorized according to molecular subgroups (WHO 2016) it seems that 1p19q-status is not a significant predictor for pre-operative epilepsy, which is also concordant with the results in our study. [5, 26, 31]

### Preoperative seizure control

In some studies, the proportion of patients with preoperative seizure control (as opposed to intractable seizures) is about 85–90% [5, 18, 32],whereas Chang et al. in 2008 reported that 132/269 (49%) in their cohort had pharmacoresistant seizures before surgery (defined as having had at least one seizure within 3 months before surgery). The short median time from diagnosis to surgery in our material, prevented useful evaluation of the preoperative seizure control rate.

### Seizure control after surgery

The proportion of seizure free patients at 1 year after surgery in our cohort was similar to that found in non-molecular series, typically reporting seizure freedom of 60–80% (range 37–82%) [6, 7, 16–19, 21, 26, 32]. There are several factors that affect results and that should be considered when interpreting seizure outcome in different studies. For example, the use of Engel class 1 by some authors [6, 16, 17] and Engel class 1a [5, 18, 19] by others is one probable factor affecting outcome. In some publications only patients with a history of seizures were included, which is also likely to impact the postoperative seizure free rates [16, 18, 19].

As seen in the meta-analysis by Bonney et al., [17] most studies on seizure control in LGG have rather short follow-up times; between 6 months to 1 year, but when longer follow-up is available it typically results in lower proportions of seizure control [21, 26]. We observed almost 10% drop in seizure freedom from 76.0 to 67.5% between 1- and 2-years postoperative (i.e., referring to patients that had been seizure free during the entire 2-year period after surgery). This effect is likely to be explained both by the natural course of the epileptic disease and possibly also by some patients experiencing tumor progression over time. [25]

#### New seizures after surgery and preoperative seizure control

The proportion of patients without a history of seizures at diagnosis, that develops seizures after surgery vary widely between studies (6–43%), as do the follow-up times in these studies (6–69 months) [6, 7, 21, 23, 26, 32].

At 1 year postoperatively, we saw that 12.9% (4/31) who initially were seizure free developed new onset seizures. At 2 years after surgery another three patients, thus cumulatively 24.1% (7/29) of those without preoperative seizures, had experienced at least one epileptic seizure (2 patients missing at 2 years).

### Factors affecting seizure outcome

#### EOR and residual tumor volume

In several studies gross total resection (GTR), as opposed to subtotal resection (STR) or biopsy, has been observed to correlate with better seizure control in LGG [4, 5, 7, 12, 22].

In the present study, we found that higher EOR significantly improved seizure status, especially for astrocytoma patients. In several recent publications on seizure outcome in histological LGG, significant associations between EOR and seizure outcome have been found [16, 18, 19]. In a study by Xu et al., an EOR threshold is suggested with better seizure outcome when EOR > 80% is achieved [16]. Still et al. reported that postoperative seizure control was more likely when EOR was ≥ 91% and/or when residual tumor volume is ≤ 19 cm^3^ in supratentorial grade 2 gliomas (according to 2007 WHO classification system) whereas Ius et al. found that seizure outcome improved when EOR ≥ 85% was obtained or when residual tumor volume was ≤ 15 cm^3^ [18, 19].

In none of the studies above the correlation between EOR/residual volume and seizure outcome has been assessed in astrocytomas and oligodendrogliomas separately. The unexpected absence of such a correlation in the oligodendroglioma cohort in the present study may be explained by our relatively small sample size—although relatively balanced between subtypes. However, it is also possible that the surgical effect on seizures is more pronounced for astrocytic tumors.

Although difficult to compare, and quite contrary to what would be expected in the case for overall survival, it is possible that EOR is a more important factor for seizure control than the highly interlinked factor of residual tumor volume. A small remnant of a large epileptogenic lesion may result in a better chance for seizure reduction than a biopsy of another (epileptogenic) tumor with size comparable to the remnant. In our material, the association to seizure outcome was more pronounced for EOR than for residual tumor volume.

#### Differing seizure mechanisms over time and between tumor subtypes?

Seizure pathogenesis in tumor associated epilepsy (TAE) is complex and not fully understood. A combination of factors such as abnormal expression of ion transporters, altered levels of amino acids in the tumor interstitium, and immunological activity is believed to cause TAE [1, 33, 34]. Mechanisms may differ between glioma types exemplified by the importance of different neurotransmitters in high- and low grade tumors where glutamate has been found to play a central role in seizure induction in high grade gliomas, whereas increased levels of the glutamate-like 2HG has been linked to seizures in IDH-mut tumors [28, 34–36] These alterations in neurotransmitter levels seem to be of main importance in the early stage of the TAE and it has been suggested that different pathogenesis may underlie seizures at presentation and later during the disease. [33, 35, 37]

Apart from tumoral and immediate peri-tumoral metabolic changes causing hyperexcitability of the cortex in diffuse glioma patients, other mechanisms such as possible alterations in synaptic plasticity and disturbance of cortico-cortical connections may cause the brain network distal to the tumor site to be affected [33]. In TAE generally, but also for oligodendrogliomas specifically, regions of cortex distant from the tumor have also been shown to be epileptiform or dysrhythmic [33, 34, 37, 38]. If the finding of this publication is valid, it is plausible that an increased propensity for oligodendrogliomas to cause seizures from such foci distant to the tumor site would explain the lesser response to EOR.

#### Tumor localization and pre-operative motor deficits

In some [5, 39] but not all [21, 22] studies, tumor localization has been found to affect seizure outcome. In a convincing study from Schucht et al. comparing postoperative seizure outcome in relation to localization, seizure freedom (Engel Class I) was achieved for only 12.1% of the 33 patients with tumors in the central area (motor and sensory strip) compared to 83.9% of the 31 patients with the frontal tumors [39]. This was irrespective of the high EOR of above 90% in both cohorts.

In the present study, postoperative seizures were more common in patients who had preoperative motor deficits or whose tumors were mainly located in the insula, the latter also in multivariable analysis and congruent with the findings in the large study by Pallud et al. [5] In the sub-analyses, the correlation between seizure outcome and tumor location was noted in univariable analyses as a tendency for worse post-operative seizure situation in oligodendrogliomas with insular location (OR 6.75; 95% CI 0.96–47.27, p= 0.054) whereas for astrocytomas, a pre-operative motor deficit was more indicative for postoperative seizures (p = 0.07; OR 5.65; 95% CI 0.85–37.46, p= 0.07).

#### Oncological therapy

In some earlier publications, postoperative therapy has been associated with better seizure outcome [23, 40–43]. We could, however, not detect any significant association between chemo-or radiotherapy within the first year of surgery and seizure freedom.

## Limitations

The study is limited by its retrospective nature and its limited sample size of 130 patients. Another limitation is the lack of data on pre-operative seizure control. This information is, however, inherently difficult to obtain in the present era, where the time between seizure onset and surgery is generally too short for evaluation of seizure control/AED effect preoperatively. Strengths of the present study include the relatively long follow-up period, the inclusion of homogeneous tumors in line with what we now define as LGG, (i.e., diffuse gliomas with *IDH*-mut) and finally that molecular sub-types were evaluated separately.

## Conclusion

In this population-based study, where all patients had *IDH*-mut LGG, we found that three out of four were seizure free (Engel Class 1A/ILAE seizure free) the first year after surgery and that a higher EOR was associated with higher probability of seizure freedom. The effect was prominent in astrocytomas, but not found in oligodendrogliomas when analyzed separately. Due to the unexpected lack of association of seizure outcome with EOR in oligodendrogliomas, further studies in the different molecular subgroups are warranted.

## Supplementary Information

Below is the link to the electronic supplementary material.

Supplementary file1 (DOCX 19 KB)

Supplementary file2 (DOCX 18 KB)

## Data Availability

The data that support the findings of the present study are not publicly available, due to them containing information that could compromise research participant privacy/consent. The data are however available upon reasonable request to the authors.

## References

[1] van Breemen MS, Wilms EB, Vecht CJ (2007). Epilepsy in patients with brain tumours: epidemiology, mechanisms, and management. Lancet Neurol.

[2] Aaronson NK, Taphoorn MJB, Heimans JJ (2011). Compromised health-related quality of life in patients with low-grade glioma. J Clin Oncol.

[3] Ruge MI, Ilmberger J, Tonn JC, Kreth F-W (2011). Health-related quality of life and cognitive functioning in adult patients with supratentorial WHO grade II glioma: status prior to therapy. J Neurooncol.

[4] Englot DJ, Berger MS, Barbaro NM, Chang EF (2011). Predictors of seizure freedom after resection of supratentorial low-grade gliomas: a review. J Neurosurg JNS.

[5] Pallud J, Audureau E, Blonski M (2014). Epileptic seizures in diffuse low-grade gliomas in adults. Brain.

[6] Chang EF, Potts MB, Keles GE (2008). Seizure characteristics and control following resection in 332 patients with low-grade gliomas. J Neurosurg.

[7] You G, Sha ZY, Yan W (2012). Seizure characteristics and outcomes in 508 Chinese adult patients undergoing primary resection of low-grade gliomas: a clinicopathological study. Neuro Oncol.

[8] Louis DN, Perry A, Reifenberger G (2016). The 2016 World Health Organization classification of tumors of the central nervous system: a summary. Acta Neuropathol.

[9] Louis DN, Perry A, Wesseling P (2021). The 2021 WHO classification of tumors of the central nervous system: a summary. Neuro Oncol.

[10] Olar A, Sulman EP (2015). Molecular markers in low-grade glioma-toward tumor reclassification. Semin Radiat Oncol.

[11] Carstam L, Corell A, Smits A (2021). WHO grade loses its prognostic value in molecularly defined diffuse lower-grade gliomas. Front Oncol.

[12] Chang EF, Smith JS, Chang SM (2008). Preoperative prognostic classification system for hemispheric low-grade gliomas in adults. J Neurosurg.

[13] Fedorov A, Beichel R, Kalpathy-Cramer J (2012). 3D Slicer as an image computing platform for the quantitative imaging network. Magn Reson Imaging.

[14] Ferreyra Vega S, Olsson Bontell T, Corell A, Smits A, Jakola AS, Carén H (2021). DNA methylation profiling for molecular classification of adult diffuse lower-grade gliomas. Clin Epigenet.

[15] Engel J (1993). Outcome with respect to epileptic seizures. Surg Treat Epilepsies.

[16] Xu DS, Awad AW, Mehalechko C (2018). An extent of resection threshold for seizure freedom in patients with low-grade gliomas. J Neurosurg.

[17] Bonney PA, Boettcher LB, Burks JD (2017). Rates of seizure freedom after surgical resection of diffuse low-grade gliomas. World Neurosurg.

[18] Still MEH, Roux A, Huberfeld G (2019). Extent of resection and residual tumor thresholds for postoperative total seizure freedom in epileptic adult patients harboring a supratentorial diffuse low-grade glioma. Neurosurgery.

[19] Ius T, Pauletto G, Tomasino B (2020). Predictors of postoperative seizure outcome in low grade glioma: from volumetric analysis to molecular stratification. Cancers (Basel).

[20] Kwan P, Arzimanoglou A, Berg AT (2010). Definition of drug resistant epilepsy: consensus proposal by the ad hoc task force of the ILAE commission on therapeutic strategies. Epilepsia.

[21] Jo J, Nevel K, Sutyla R, Smolkin M, Lopes MB, Schiff D (2021). Predictors of early, recurrent, and intractable seizures in low-grade glioma. Neurooncol Pract.

[22] Shan X, Fan X, Liu X, Zhao Z, Wang Y, Jiang T (2018). Clinical characteristics associated with postoperative seizure control in adult low-grade gliomas: a systematic review and meta-analysis. Neuro Oncol.

[23] Kahlenberg CA, Fadul CE, Roberts DW (2012). Seizure prognosis of patients with low-grade tumors. Seizure.

[24] Hajian-Tilaki K (2013). Receiver operating characteristic (ROC) curve analysis for medical diagnostic test evaluation. Caspian J Intern Med Spring.

[25] Santos-Pinheiro F, Park M, Liu D (2019). Seizure burden pre- and postresection of low-grade gliomas as a predictor of tumor progression in low-grade gliomas. Neurooncol Pract.

[26] Roberts M, Northmore T, Shires J, Taylor P, Hayhurst C (2018). Diffuse low grade glioma after the 2016 WHO update, seizure characteristics, imaging correlates and outcomes. Clin Neurol Neurosurg.

[27] Zhong Z, Wang Z, Wang Y, You G, Jiang T (2015). IDH1/2 mutation is associated with seizure as an initial symptom in low-grade glioma: a report of 311 Chinese adult glioma patients. Epilepsy Res.

[28] Liubinas SV, D'Abaco GM, Moffat BM (2014). IDH1 mutation is associated with seizures and protoplasmic subtype in patients with low-grade gliomas. Epilepsia.

[29] Stockhammer F, Misch M, Helms HJ (2012). IDH1/2 mutations in WHO grade II astrocytomas associated with localization and seizure as the initial symptom. Seizure.

[30] Yang Y, Mao Q, Wang X (2016). An analysis of 170 glioma patients and systematic review to investigate the association between IDH-1 mutations and preoperative glioma-related epilepsy. J Clin Neurosci.

[31] Mulligan L, Ryan E, O'Brien M (2014). Genetic features of oligodendrogliomas and presence of seizures. The relationship of seizures and genetics in LGOs. Clin Neuropathol.

[32] You G, Huang L, Yang P (2012). Clinical and molecular genetic factors affecting postoperative seizure control of 183 Chinese adult patients with low-grade gliomas. Eur J Neurol.

[33] Beaumont A, Whittle IR (2000). The pathogenesis of tumour associated epilepsy. Acta Neurochir (Wien).

[34] Armstrong TS, Grant R, Gilbert MR, Lee JW, Norden AD (2016). Epilepsy in glioma patients: mechanisms, management, and impact of anticonvulsant therapy. Neuro Oncol.

[35] Sørensen MF, Heimisdóttir SB, Sørensen MD (2018). High expression of cystine-glutamate antiporter xCT (SLC7A11) is an independent biomarker for epileptic seizures at diagnosis in glioma. J Neurooncol.

[36] Chen H, Judkins J, Thomas C (2017). Mutant IDH1 and seizures in patients with glioma. Neurology.

[37] Kerkhof M, Benit C, Duran-Pena A, Vecht CJ (2015). Seizures in oligodendroglial tumors. CNS Oncol.

[38] Whittle IR, Beaumont A (1995). Seizures in patients with supratentorial oligodendroglial tumours. Clinicopathological features and management considerations. Acta Neurochir (Wien).

[39] Schucht P, Ghareeb F, Duffau H (2013). Surgery for low-grade glioma infiltrating the central cerebral region: location as a predictive factor for neurological deficit, epileptological outcome, and quality of life. J Neurosurg.

[40] Koekkoek JA, Dirven L, Heimans JJ (2015). Seizure reduction in a low-grade glioma: more than a beneficial side effect of temozolomide. J Neurol Neurosurg Psychiatry.

[41] Sherman JH, Moldovan K, Yeoh HK (2011). Impact of temozolomide chemotherapy on seizure frequency in patients with low-grade gliomas. J Neurosurg.

[42] Rudà R, Magliola U, Bertero L (2013). Seizure control following radiotherapy in patients with diffuse gliomas: a retrospective study. Neuro Oncol.

[43] van den Bent MJ, Afra D, de Witte O (2005). Long-term efficacy of early versus delayed radiotherapy for low-grade astrocytoma and oligodendroglioma in adults: the EORTC 22845 randomised trial. Lancet.

