# Supplementary material for: Seizures in patients with IDH-mutated lower grade gliomas
Source: J Neurooncol. 2022 Oct 18;160(2):403–11. doi: 10.1007/s11060-022-04158-6 (PMC9722876; doi:10.1007/s11060-022-04158-6)
Supplement: Supplementary file 1 — Supplementary file1 (DOCX 19 KB) [file 11060_2022_4158_MOESM1_ESM.docx]

**Supplementary Table 1**

**Seizures in patients with *IDH*-mutated lower grade gliomas**

**Journal of Neuro-Oncology**

Louise Carstam^,^ Isabelle Rydén and Asgeir Store Jakola^,^

Department of Neurosurgery, Sahlgrenska University Hospital, Göteborg, Sweden

**Corresponding author**:

Louise Carstam, MD

E-mail: Louisecarstam@hotmail.com

| Supplementary Table 1  Predictors for postoperative epileptic seizures within 12 months after surgery in astrocytoma patients according to unadjusted and adjusted analysis. N=67 | | | | | | | |
| --- | --- | --- | --- | --- | --- | --- | --- |
| Variable | | Univariable analysis | | | Multivariable analysis | | |
|  |  | Unadjusted Odds  Ratio | 95% CI | p-value | Adjusted  Odds  Ratio | 95% CI | p-value |
| Sex | Female  Male | 1 (ref)  0.76 | 0.25-2.34 | 0.63 |  |  |  |
| Age | per year | 0.98 | 0.94-1.03 | 0.46 |  |  |  |
| Preop tumor volume | per cm^3^ | 1.01 | 1,00-1.02 | **0.02** |  |  | * |
| Preop motor deficit | No  Yes | 1 (ref)  5.65 | 0.85-37.46 | 0.07 | 3.94 | 0.53-29.37 | 0.18 |
| WHO grade | Grade 2  Grade 3 | 1 (ref)  1.04 | 0.34-3.20 | 0.95 |  |  |  |
| Location of tumor | Frontal  Temporal  Insular  Other | 1 (ref)  0.38  6.0  1.29 | 0.07-1.96  0.49-74.29  0.27-6.05 | 0.25  0.16  0.75 |  |  |  |
| Residual tumor volume | per cm^3^ | 1.01 | 1.00-1.02 | **0.03** |  |  | ***** |
| EOR | per unit | 0.97 | 0.96-0.99 | **<0.01** | 0.98 | 0.96-0.99 | **<0.01** |
| Chemotherapy within 12 months postop | No  Yes | 1 (ref)  1.25 | 0.35-4.50 | 0.73 |  |  |  |
| Radiotherapy within 12 months postop | No  Yes | 1 (ref)  1.33 | 0.32-5.47 | 0.69 |  |  |  |

EOR = Extent of resection

* Not included in multivariable analysis due to significant correlation with EOR
