# Supplementary material for: Seizures in patients with IDH-mutated lower grade gliomas
Source: J Neurooncol. 2022 Oct 18;160(2):403–11. doi: 10.1007/s11060-022-04158-6 (PMC9722876; doi:10.1007/s11060-022-04158-6)
Supplement: Supplementary file 2 — Supplementary file2 (DOCX 18 KB) [file 11060_2022_4158_MOESM2_ESM.docx]

**Supplementary Table 2**

**Seizures in patients with *IDH*-mutated lower grade gliomas**

**Journal of Neuro-Oncology**

Louise Carstam^,^ Isabelle Rydén and Asgeir Store Jakola^,^

Department of Neurosurgery, Sahlgrenska University Hospital, Göteborg, Sweden

**Corresponding author**:

Louise Carstam, MD

E-mail: Louisecarstam@hotmail.com

| Supplementary Table 2  Predictors for postoperative epileptic seizures within 12 months after surgery in oligodendroglioma patients. N=62 | | | | | | | |
| --- | --- | --- | --- | --- | --- | --- | --- |
| Variable | | Univariable analysis | | | Multivariable analysis | | |
|  |  | Unadjusted Odds  Ratio | 95% CI | p-value | Adjusted  Odds  Ratio | 95% CI | p-value |
| Sex | Female  Male | 1 (ref)  0.54 | 0.17-1.75 | 0.31 |  |  |  |
| Age | per year | 0.98 | 0.94-1.03 | 0.49 |  |  |  |
| Preop tumor volume | per | 1.00 | 0.99-1.01 | 0.45 |  |  |  |
| Preop motor deficit | No  Yes | 1 (ref)  3.46 | 0.44-27.02 | 0.24 |  |  |  |
| WHO grade | grade 2  grade 3 | 1 (ref)  0.44 | 0.13-1.49 | 0.19 |  |  |  |
| Location of tumor | frontal  temporal  insular  other | 1 (ref)  2.25  6.75  1.80 | 0.35-14.49  0.96-47.27  0.29-11.00 | 0.93  0.054  0.52 |  |  |  |
| Residual tumor volume | per ml | 1.00 | 0.99-1.01 | 0.95 |  |  |  |
| EOR | per unit | 0.99 | 0.97-1.01 | 0.34 |  |  |  |
| Chemotherapy within 12 months postop | No  Yes | 1 (ref)  1.13 | 0.33-3.87 | 0.84 |  |  |  |
| Radiotherapy within 12 months postop | No  Yes | 1 (ref)  1.36 | 0.40-4.60 | 0.62 |  |  |  |

EOR = Extent of resection
